# *Ascophyllum nodosum* based plant biostimulant shapes the bacterial community in the rhizosphere of corn

**DOI:** 10.1186/s12870-025-07270-7

**Published:** 2025-10-01

**Authors:** Vinod Kumar Selvaraj, Jai Singh Patel, Joseph P.M. Hui, Junzeng Zhang, Fabrice Berrué, Balakrishnan Prithiviraj

**Affiliations:** 1 Department of Plant, Food and Environmental Sciences, Faculty of Agriculture, Dalhousie University, Truro, Nova Scotia, B2N 5E3 Canada; 2 Aquatic and Crop Resource Development Research Centre, National Research Council of Canada, Nova Scotia B3H 3Z1 Halifax, Canada; 3 Department of Mycology and Plant Pathology, Institute of Agricultural Sciences, Banaras Hindu University, Varanasi, 221005 Uttar Pradesh India

**Keywords:** *Ascophyllum nodosum*, Root exudates, *Pseudomonas protegens* (CHA0), 2,4-dihydroxy-7-methoxy-1,4-benzoxazin-3-one (DIMBOA), 6-methoxybenzoxazolinone (MBOA)

## Abstract

**Background:**

Plant biostimulants are an emerging class of agricultural inputs known to enhance plant growth and improve their tolerance to abiotic stress. While biostimulants are widely used, their mechanisms of action remain poorly understood. This study investigates the effects of an *Ascophyllum nodosum*-based biostimulant (ANE) on the rhizosphere bacterial communities of corn (*Zea mays*).

**Results:**

Root exudates from ANE root-treated plants promoted the swarming motility of *Pseudomonas protegens* (CHA0), a plant growth-promoting rhizobacterium. Gene expression analysis showed that root exudates from 0.01% ANE-treated plants up-regulated *P. protegens* CHA0 genes associated with chemotaxis (*cheW*, *cheV*), pyoverdine (*pvdS*), pyrrolnitrin (*prnD*), and hydrogen cyanide (*hcnA*) biosynthesis compared to controls. ANE also significantly altered rhizosphere microbiome composition, increasing the abundance of genera such as *Chryseolinea*, *Pseudoxanthomonas*, *Novosphingobium*, *Quadrisphaera*, *Turneriella*, and *Kitasatospora*. Liquid chromatography-high resolution mass spectrometry (LC–HRMS) and partial least squares-discriminant analysis (PLS-DA) revealed distinct chemical profiles in the root extracts of ANE-treated plants. Specifically, ANE increased the concentrations of benzoxazinoids, including 2,4-dihydroxy-7-methoxy-1,4-benzoxazin-3-one (DIMBOA) and 6-methoxybenzoxazolinone (MBOA) in maize roots by approximately 1.4-fold and 1.76-fold, respectively.

**Conclusion:**

Overall, these findings suggest that ANE modifies the rhizosphere microbiome by influencing the chemical composition of both root tissue and root exudates.

**Graphical abstract:**

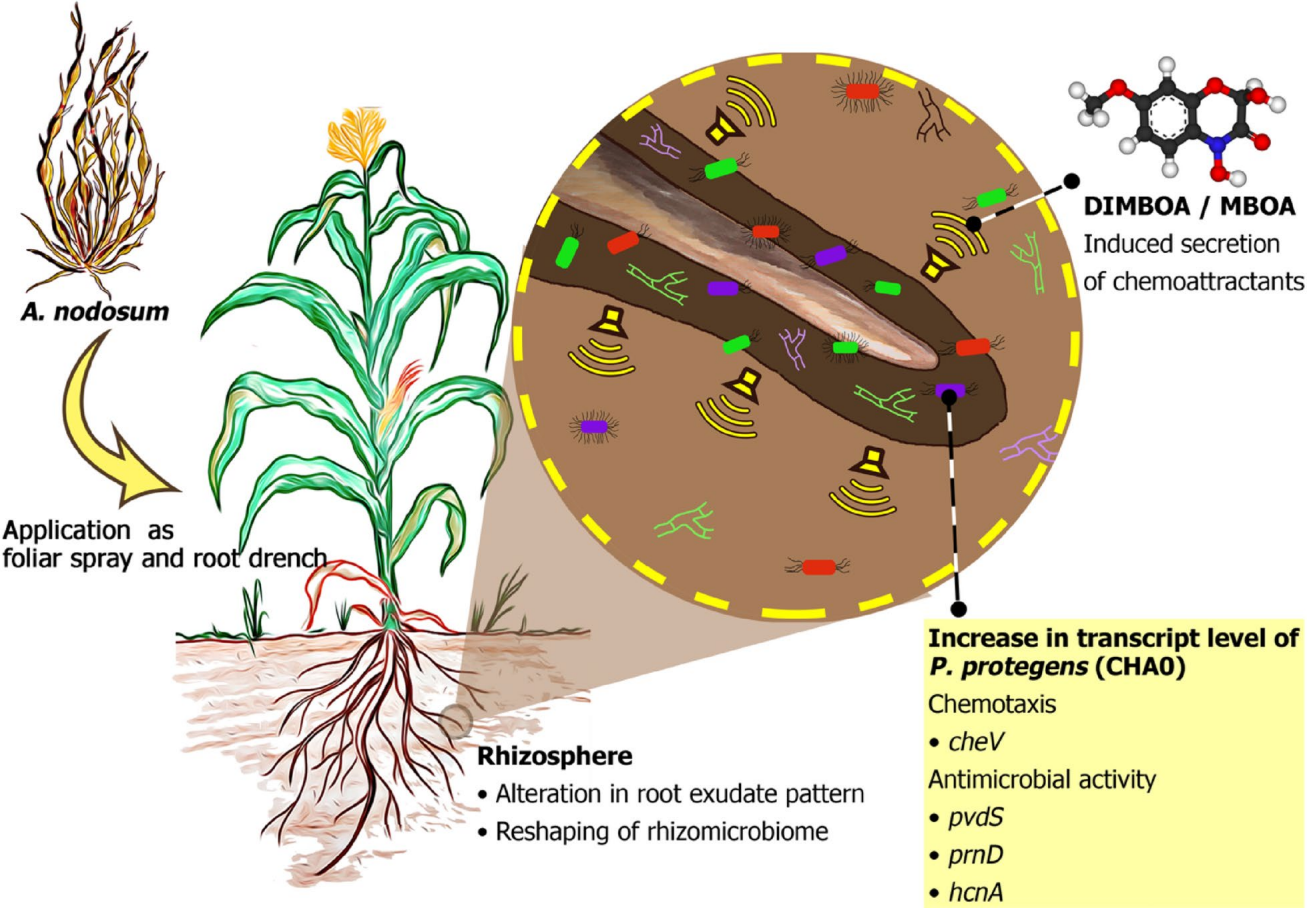

**Supplementary Information:**

The online version contains supplementary material available at 10.1186/s12870-025-07270-7.

## Background

Global food demand is rising as the world’s population is projected to reach 9.7 billion by 2050 [1]. Ensuring adequate plant food production is crucial to meet this demand. While synthetic fertilizers and pesticides have been widely used to enhance food production, their detrimental effects on the environment, animals, and human health are well documented [2, 3]. Additionally, long-term use of inorganic fertilizers significantly alters the population structure, diversity, and activity of soil microbial communities [4]. To mitigate these negative effects, the use of beneficial microbes, such as microbial biocontrol agents and plant growth-promoting bacteria [PGPB], is gaining attention as an alternative approach.

The rhizomicrobiome, the community of microorganisms surrounding plant roots, plays an integral role in the soil-plant system, influencing plant development and health [5]. The rhizomicrobiome promotes plant growth through processes such as organic matter decomposition, nutrient mobilization, nitrogen fixation, and phosphate and potassium mineralization, contributing to crop protection, production, and productivity [6]. It also regulates biogeochemical processes that affect soil nutrient and organic composition [7]. Rhizomicrobiome composition is strongly influenced by rhizodeposition, which includes root exudates, volatile compounds, and lysates [8]. Root exudates, in particular, shape rhizosphere microorganisms by providing nutrients and promoting chemotaxis through chemo-attractants [9–11]. These exudates are complex, often containing phenolics, organic acids, amino acids, vitamins, purines, and enzymes [12, 13].

PGPBs accelerate plant growth by secreting extracellular metabolites such as gibberellins, cytokinins, and indole acetic acid [14]. Additionally, their colonization of the rhizosphere and secretion of secondary metabolites stimulate root hair and lateral root formation, enhancing water and nutrient uptake [15]. *Bacillus* species, for example, have been shown to improve seed germination, seedling vigor, and nitrogen content in cucumber plants [16], suppress disease, and promote plant growth in carnations and cotton [17, 18]. PGPBs also play a key role in enhancing abiotic stress tolerance in plants. For example, various microbes including, *P. putida* [19], *Azospirillum* spp. [20], *B. thuringiensis* [21], *B. megaterium* and *Rhizophagus intraradices* [22], improve drought tolerance by enhancing photosynthesis, water conductivity, expression of water transporter genes, and oxidative phosphorylation, while reducing stomatal conductance and lipid oxidation. Despite promising results, the application of beneficial microbes in field conditions remains challenging due to variable environmental and experimental conditions. For example, while *Bacillus* spp. showed biocontrol activity against potato pathogens *in vitro*, its performance declined under chaotropic conditions, allowing pathogen proliferation [23].

Seaweeds, marine macroalgae classified into red (Rhodophyta), brown (Phaeophyta), and green (Chlorophyta) groups, are widely used as plant biostimulants [24]. The European Biostimulant Industry Council (EBIC) defines biostimulants as products containing substances or microorganisms that stimulate natural processes to enhance nutrient uptake, nutrient efficiency, abiotic stress tolerance, and crop quality [25]. Among seaweeds, brown algae, particularly *Ascophyllum nodosum*, are commonly used in agriculture due to their biostimulant properties [26]. *A. nodosum* extracts have been shown to promote plant growth and tolerance to a wide range of abiotic stresses, including salinity [27, 28] and drought [29–32], across various plant species. Treatment with *A. nodosum* extracts conferred tolerance to salt [33, 34], freezing [35], and drought [36] in Arabidopsis and reduced pod shatter and yield loss in commercially cultivated rapeseed [37]. Additionally, *A. nodosum*-based biostimulants enhanced plant resistance to several fungal and bacterial pathogens [27, 28, 38]. Application of *A. nodosum* extract in tomato, revealed the up-regulation of 635 and down-regulation of 456 genes. Further, KEGG pathway analysis revealed that the treatment influenced the expression of genes related to growth promotion and plant-pathogen interactions [39]. ANE applications to tomato crop promoted plant growth, yield parameters, and also improved the soil fertility. The study also emphasized soil application of ANE was much effective than foliar spray [40]. In another recent study, ANE application to tomato improved the fruit quality traits including, total soluble solids, ascorbic acid content, lycopene, and total sugars than the titratable acidity content [41]. However, the exact modes of action remain unclear, likely due to the complex chemical composition of the extracts [42]. Despite numerous studies on *A. nodosum*-based biostimulants (ANE) and their effects on plant growth and biotic/abiotic stress tolerance, their precise modes of action remain poorly understood, particularly in commercially cultivated crops. In this study, we investigated the effects of root exudates from ANE-treated maize plants on physiology and motility of *Pseudomonas protegens* (CHA0). We also analyzed the soil rhizobacterial community following ANE treatment and examined how ANE alters the root exudate profile in maize plants. Further, we have treated the plant with ANE as soil drench/root treatment and foliar spray separately to observe the indirect effect on the root system of maize plant by applying ANE as a foliar spray. Our findings shed light on the indirect modes of action of ANE, particularly its ability to modify root exudate patterns and shape the rhizomicrobiome, an underexplored potential of biostimulants.

## Methods

### Plant material, growth condition, treatment, and sampling

Preparation of plants: Sweet corn seeds (hybrid-Bi-Licious, Halifax Seeds, Canada) were surface-sterilized using 0.5% sodium hypochlorite, thoroughly rinsed with sterile distilled water, and soaked for 12 h. Once fully imbibed, the seeds were germinated on sterile trays by incubating in the dark at 30 ± 2 °C for 5 days.

Experiments were conducted in two growth media including, hydroponics and soil.

Hydroponics experiment: Plant Culture: Uniformly germinated seedlings were transferred to magenta jars containing sterile, half-strength Hoagland solution (Phytotechnology Laboratories, USA), with polystyrene weigh boats acting as covers (with a central hole for the plants). The seedlings were incubated in a growth chamber (Conviron, Canada) for 10 days at 28 ± 2 °C under a 16-hour light/8-hour dark cycle.

Preparation of treatment solution: The *Ascophyllum nodosum* extract (ANE) powder, an alkaline extract, was sourced from Acadian Seaplants Ltd., Canada. This extract was dissolved in sterile water to achieve the desired concentrations, with 0.1% Silwet (PhytoTech, USA) added as a surfactant.

Treatment: ANE treatments were administered via either foliar spray (FS) or root treatment (RT). Root treatments involved adding filter-sterilized ANE to the Hoagland solution (HS) at two concentrations: 0.001% ANE (T1-ANE RT 0.001%) and 0.01% ANE (T2-ANE RT 0.01%)). Foliar treatments utilized 0.01% ANE (T3-ANE FS 0.01%) and 0.1% ANE (T4-ANE FS 0.1%), with approximately 5 ml of the solution sprayed onto each plant to completely coat the leaves, while ensuring no contact between the ANE and the roots (by covering the base with plastic sheeting). Control groups included untreated plants (T5-Untreated plant), Hoagland solution supplemented with ANE at 0.001% (T1C-ANE 0.001%) or 0.01% (T2C-ANE 0.01%) without plants, and a Hoagland solution control (T5C-HS). The experiment was replicated three times, with 20 plants per treatment in each replication.

Sampling: Fifteen days post-treatment, plants were harvested from the magenta jars. Root exudates, along with the growth solution, were tested for bacterial contamination by plating on LB media. Contaminant-free solutions were stored at −20 °C for further biological and chemical analyses. Roots were also collected and stored at −20 °C for further root exudate analysis.

Soil experiment: Preparation of soil: For the soil experiment, topsoil (0–15 cm) was collected from the demonstration garden at the Faculty of Agriculture, Dalhousie University (Truro, Canada). This soil was sieved to uniform consistency and packed into SC-10 containers (164 ml capacity). Raising of plants: Maize seedlings were transferred to the containers and incubated in a glasshouse (Conviron, Canada) at 28 ± 2 °C under a 16-hour light/8-hour dark cycle.

Treatment: After 10 days, the plants were treated with ANE either via soil drenching or foliar spraying. Soil drenches were performed using 0.001% (T1-ANE RT 0.001%) and 0.01% (T2-ANE RT 0.01%) ANE solutions, applying 20 ml to saturate the entire soil column. Treatments were repeated twice at 7-day intervals. Foliar treatments were conducted similarly to the hydroponics experiment using 0.01% ANE (T3-ANE FS 0.01%) and 0.1% ANE (T4-ANE FS 0.1%). Control groups included untreated plants (T5-Untreated Plant), soil treated with ANE without plants (T1C-ANE 0.001% and T2C-ANE RT 0.01%), and untreated soil without plants (T5C-Soil). The experiment was replicated three times, with 10 plants per treatment in each replication.

Sampling: Soil samples were collected one week after the second treatment, with root-adhering soil gently extracted for analysis. Soil sampling was conducted in two ways: (1) rhizosphere soil adhering to roots was collected for colony-forming unit (CFU) counts and root exudate analysis, and (2) soil from the top and bottom zones was collected separately for bacterial community population analysis using amplicon sequencing.

### Swim plate assay

Root exudates from hydroponics-grown plants were freeze-dried, pooled (5 plants per replicate), and reconstituted to 5 ml with sterile distilled water. Swim plates were prepared with LB amended with 0.3% agar [43]. For the swim plate assay, 5 ml of the *Pseudomonas protegens* (CHA0) overnight culture (OD = 0.1) was inoculated into one corner of the plate, while a sterile filter paper disc loaded with 100 µl of root exudate was placed on the opposite corner. Bacterial movement was recorded after 12 and 24 h. The experiment was performed three times, with three replicates each time.

### Quantitative RT-PCR (qRT-PCR) analysis

*Pseudomonas protegens* (CHA0) was cultured in LB broth at 28 ± 2 °C with 200 rpm shaking. After 6 h, cells were harvested by low-speed centrifugation, resuspended in 2 ml sterile distilled water to an OD of 0.1, and incubated with 1 ml of root exudate solution (prepared similar to the swim plate assay) from various treatments for 3 h. RNA was stabilized using RNA protect cell reagent (Qiagen, USA), isolated with the RNeasy Mini Kit (Qiagen, USA), and quantified using NanoDrop (NanoDrop™ 2000, Thermo Fisher Scientific). cDNA was synthesized using the RevertAid RT Reverse Transcription Kit (Thermo Fisher Scientific, USA).

Expression levels of genes related to chemotaxis (*cheA*,* cheW*,* cheV*), pyoverdine synthesis (*pvdS*), pyrrolnitrin (*prnD*), and hydrogen cyanide (*hcnA*) were assessed in *P. protegens* (CHA0) using real-time qRT-PCR (QuantStudio-3, Thermo Fisher Scientific) and SYBR^®^ Green Supermix Kit (Bio-Rad, USA). Transcript levels were normalized to the internal control gene *rpoC*. Primer sequences (Table S1) were designed using Primer3 software (v4.1.0). Three biological replicates with three technical replicates were performed.

### Profiling of rhizosphere soil bacterial community

Rhizosphere soil samples were serially diluted, and CFU counts for bacteria was estimated. Five biological replicates with two technical replicates were used. DNA from each soil sample was extracted using the E.Z.N.A. Soil DNA Kit (Omega Bio-Tek, USA), quantified using NanoDrop, and subjected to 16 S rRNA (V3/V4 regions) sequencing (Integrated Microbiome Resource, Dalhousie University). Data analysis was performed using STAMP v2.1.3 [44], and the Weighted Unifrac of the bacterial community was assessed using Emperor ordination in Qiime2view.

### Analyses of root exudates, root and rhizosphere soil extracts by liquid Chromatography-High resolution mass spectrometry (LC–HRMS)

#### Samples Preparation

For root extract preparation, roots were ground in liquid nitrogen, and 2 g of the resulting powder was extracted twice with 5 ml HPLC-grade methanol. The extract was centrifuged to remove debris, filtered (0.25 μm membrane), and three biological replicates were collected per treatment.

Root exudate samples were desalted using C18-SPE columns (Sigma-Aldrich, USA) following standard protocol [45]. Methanol with 2% formic acid and MilliQ water were used for washing and elution, and four replicates per treatment were prepared.

For soil extract preparation, 5 g of rhizosphere soil was extracted twice with 5 ml methanol-water (1:1 v/v), centrifuged, and supernatants combined for analysis [29]. Three biological replicates per treatment were collected.

#### Untargeted LC-HRMS analysis

The analysis was carried out using a LC-HRMS system (Thermo Fisher Scientific, Waltham, MA), coupled with an Exactive high-resolution mass spectrometer that was equipped with an electrospray ionization source. Separation was performed on an Acquity HSS T3 column (2.1 × 100 mm, 1.8 μm, Waters, Milford, MA, USA). The solvent comprised of 0.1% formic acid in deionized water (A) and 0.1% formic acid in acetonitrile (B) at 0.35 ml/min, with a linear gradient from 5 to 100% B in 4 min. MS acquisition was performed in both positive and negative polarities at a scan rate of 4 Hz. The ion source conditions consist of spray voltage of 2.5 kV, sheath flow of 50, the auxiliary flow of 10, and capillary and heater temperatures of 360 °C and 300 °C, respectively. The root extract and rhizosphere soil extracts (in 10 mL methanol) were filtered through a Durapore PVDF 0.22 μm filter (Millipore) and dried using a centrifugal evaporator (Genevac, SP Scientific, Warminster, PA, USA). They were finally dissolved in 200 µL MeOH, and 10-fold diluted samples were used for LC-HRMS analysis. Root exudate samples were dried as described above and dissolved in 200 µL methanol for LC-HRMS analysis. Duplicate injections were used for the untargeted analysis.

For data analysis, the LC-HRMS raw data files were converted to netCDF files using a built-in software module XCalibur (Thermo Fisher Scientific, Waltham, MA, USA), and then an open source software mzMine 2 (http://mzmine.github.io/) was employed for preprocessing, including mass detection, chromatogram building, deisotoping, and joint alignment. The list of buckets defined by a retention time (Rt) and mass to charge ratio (m/z) was compared between sample datasets using multivariate data analysis software SIMCA-*P* + 12.0.1 (Sartorius Stedim Data Analytics AB, Umea, Sweden) to identify key discriminative ions in the datasets. The key metabolites were identified based on accurate mass, isotope peak pattern, fragmentation, plant metabolomics and other natural products database, and relevant information in the literature.

### Quantification of benzoxazinoids (DIMBOA and MBOA)

To confirm the identified masses of DIMBOA and MBOA from the untargeted LC-HRMS analysis, standards were procured from Sigma-Aldrich, USA (MBOA), and Toronto Research Chemicals, Canada (DIMBOA). Quantification was carried out across treatments using the same LC-HRMS system as described earlier. Stock solutions of MBOA and DIMBOA (10 mM) were prepared in methanol, and a dilution series was created with concentrations ranging from 0.1 µM to 100 µM.

DIMBOA and MBOA in the roots, root exudate and rhizosphere soil extracts were quantified. Among all the samples the root extracts were detected with higher quantity of DIMBOA and MBOA and the results were very consistent. Consequently, only root extracts were further analyzed and quantified using a new set of samples. The root extracts were prepared in 10 ml methanol (as described previously) and injected directly into the LC-HRMS system for analysis. Accurate masses of MBOA and DIMBOA were used to extract their peak areas within a mass window of 5 ppm, and their concentrations were calculated based on these peak areas.

### Statistical analysis

Data were analyzed with SPSS statistics (ver 25.0.0.1). One-way Analysis of Variance (ANOVA) analyses was performed followed by Duncan’s multiple range test (*p* value ≤ 0.05).

## Results

### Root exudates of the ANE-treated plants induced the swarming motility of P. protegens (CHA0)

**Fig. 1:**
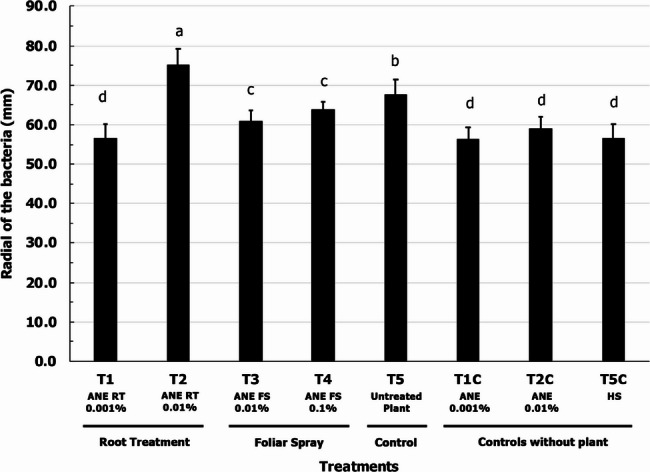
Swarming motility of *P. protegens* (CHA0) towards root exudates from maize plants treated with ANE. T1 – Root treatment with 0.001% ANE; T2 - Root treatment with 0.01% ANE; T3 – Foliar spray with 0.01% ANE; T4 - Foliar spray with 0.1% ANE; T5 – Untreated control; T6–0.001% ANE; T7−0.01% ANE; Control – Water control. Data are represented as mm. Error bars indicate standard deviation obtained from three replicates per treatment. Analysis of variance was performed through DMRT with SPSS statistics (version 25, IBM). Means followed by common letters are not significantly different

To investigate the effect of ANE on the swarming motility of *P. protegens* (CHA0), a swim plate assay was conducted using root exudates (hereafter referred to as “exudates”) collected from corn plants treated with ANE. As illustrated in Fig. 1, the bacteria exhibited faster movement towards exudates from root-treated plants with 0.01% ANE (T2-ANE RT 0.01%). The bacterial movement reached up to 75 mm, whereas the movement in plates containing exudates from untreated plants (T5-Untreated Plant) was 68 mm.

In contrast, exudates from foliar-treated plants with varying concentrations of ANE (T3-ANE FS 0.01% & T4-ANE FS 0.1%) did not enhance bacterial movement and showed lower attraction compared to the untreated control (T5-Untreated Plant). Furthermore, direct treatment with ANE in the absence of exudates (T1C-ANE 0.001% and T2C-ANE RT 0.01%)) did not demonstrate significant effects, remaining similar to the control treatment.

These findings suggest that ANE, when applied to the roots, stimulates the plants to release compounds that likely attract *P. protegens* CHA0.

### The effect of root exudate on the P. protegens (CHA0) gene expression

**Fig. 2:**
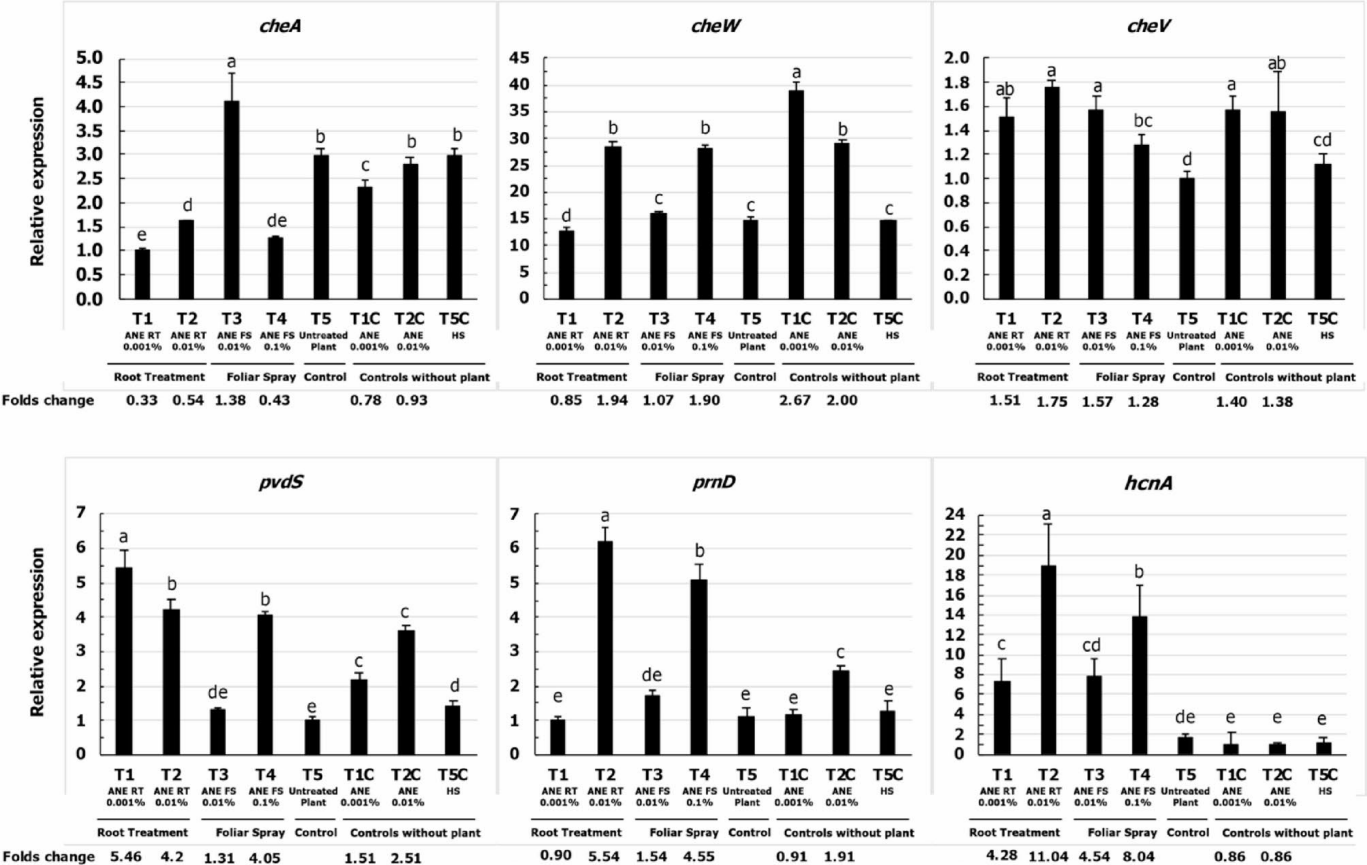
Relative expression of key genes in *P. protegens* (CHA0) treated with root exudates from maize plants treated with ANE. T1 – Root treatment with 0.001% ANE; T2 - Root treatment with 0.01% ANE; T3 – Foliar spray with 0.01% ANE; T4 - Foliar spray with 0.1% ANE; T5 – Untreated control; T1C - ANE 0.001% (no root exudate); T2C - ANE 0.01% (no root exudate); T5C – ½ strength Hoaglands solution (no root exudate). Error bars indicate standard deviation obtained from three replicates per treatment. Analysis of variance was performed through DMRT with SPSS statistics (version 25, IBM). Means followed by common letters are not significantly different

The expression levels of genes involved in chemotaxis (*cheA*,* cheW*,* cheV*), pyoverdine synthesis (*pvdS*), pyrrolnitrin production (*prnD*), and hydrogen cyanide synthesis (*hcnA*) were analyzed in *P. protegens* (CHA0) in response to root exudates and ANE treatments. As shown in Fig. 2, *cheW* was the most strongly induced gene among the chemotaxis-related genes. Its expression was increased by up to 2-fold in response to T2-ANE RT 0.01%,(root treatment with 0.01% ANE) and T4-ANE FS 0.1%,(foliar spray with 0.1% ANE) compared to T5-Untreated plant. Treatment with only ANE, T1C-ANE 0.001% and T2C-ANE RT 0.01% also elevated *cheW* transcript levels by 2 to 2.7-fold compared to the Hoagland solution control (T5C-HS).

For *cheV*, there was a slight up-regulation with both root exudates and ANE treatments. The expression was induced by 0.3 to 0.75-fold across all the treatments compared to their respective controls. *cheA* expression was induced up to 1.38-fold in T3-ANE FS 0.01%, (root exudates from plants foliar-treated with 0.01% ANE) compared to T5-Untreated plant. However, all the other slightly down-regulated *cheA* expression.

The expression of *pvdS* was enhanced in all the treatments, with an induction of 4 to 5.4-fold observed in T1-ANE RT 0.001% (root treatment with 0.001% ANE), T2-ANE RT 0.01% (root treatment with 0.01% ANE), and T4-ANE FS 0.1% (foliar-treated with 0.1% ANE) compared to T5-Untreated plant. Direct ANE treatments without plants, T1C-ANE 0.001% and T2C-ANE 0.01% also up-regulated *pvdS* expression. Similarly, *hcnA* was significantly up-regulated by the root exudates from all the treatments, with T2-ANE RT 0.01% (root treatment with 0.01% ANE) showing an 11-fold induction, the highest expression observed for *hcnA*. However, direct ANE treatments without plants, T1C-ANE 0.001% and T2C-ANE 0.01% did not significantly affect *hcnA* expression.

The *prnD* gene was induced specifically by T2-ANE RT 0.01%(root treatment with 0.01% ANE) and T4-ANE FS 0.1% (foliar treatment with 0.1% ANE), with a 5.5 and 4.5-fold increase, respectively compared to T5-Untreated plant. Direct ANE treatments without plants T2C-ANE 0.01% also increased *prnD* expression by 2-fold compared to Hoagland solution control (T5C-HS).

Overall, T2-ANE RT 0.01% (root treatment with 0.01% ANE) was the most effective in inducing the expression of genes in *P. protegens* CHA0 related to chemotaxis and antimicrobial activity. Additionally, some genes were modulated by direct ANE application, but these effects were less pronounced compared to the influence of root exudates. Our results indicate that ANE influences the expression of key genes in *P. protegens* both directly and indirectly, with root exudate-mediated effects being more prominent.

### ANE treatment significantly influenced the rhizosphere soil bacterial community profile

**Fig. 3:**
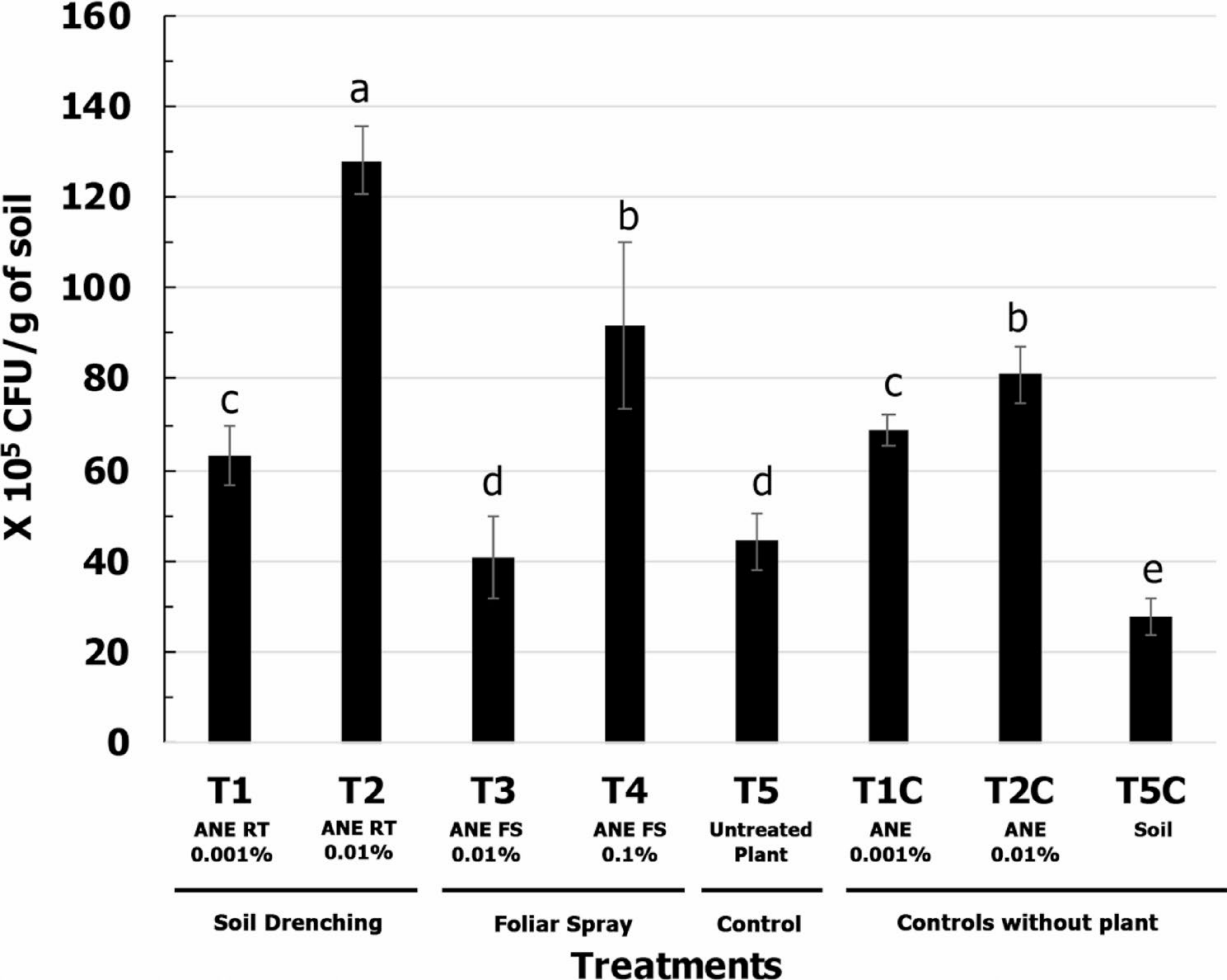
Population of culturable bacterial community in the maize, rhizosphere, soil treated with ANE. Data are represented as X10^5^ CFU/g of soil. T1 – Soil drench with 0.001% ANE; T2 - Soil drench with 0.01% ANE; T3 – Foliar spray with 0.01% ANE; T4 - Foliar spray with 0.1% ANE; T5 – Untreated control; T5C – untreated soil without plant; T1C - Soil drench ANE 0.001% without plant; T2C - Soil drench ANE 0.01% without plant. Error bars indicate standard deviation obtained from five replicates per treatment. Analysis of variance was performed through DMRT with SPSS statistics (version 25, IBM). Means followed by common letters are not significantly different

**Fig. 4:**
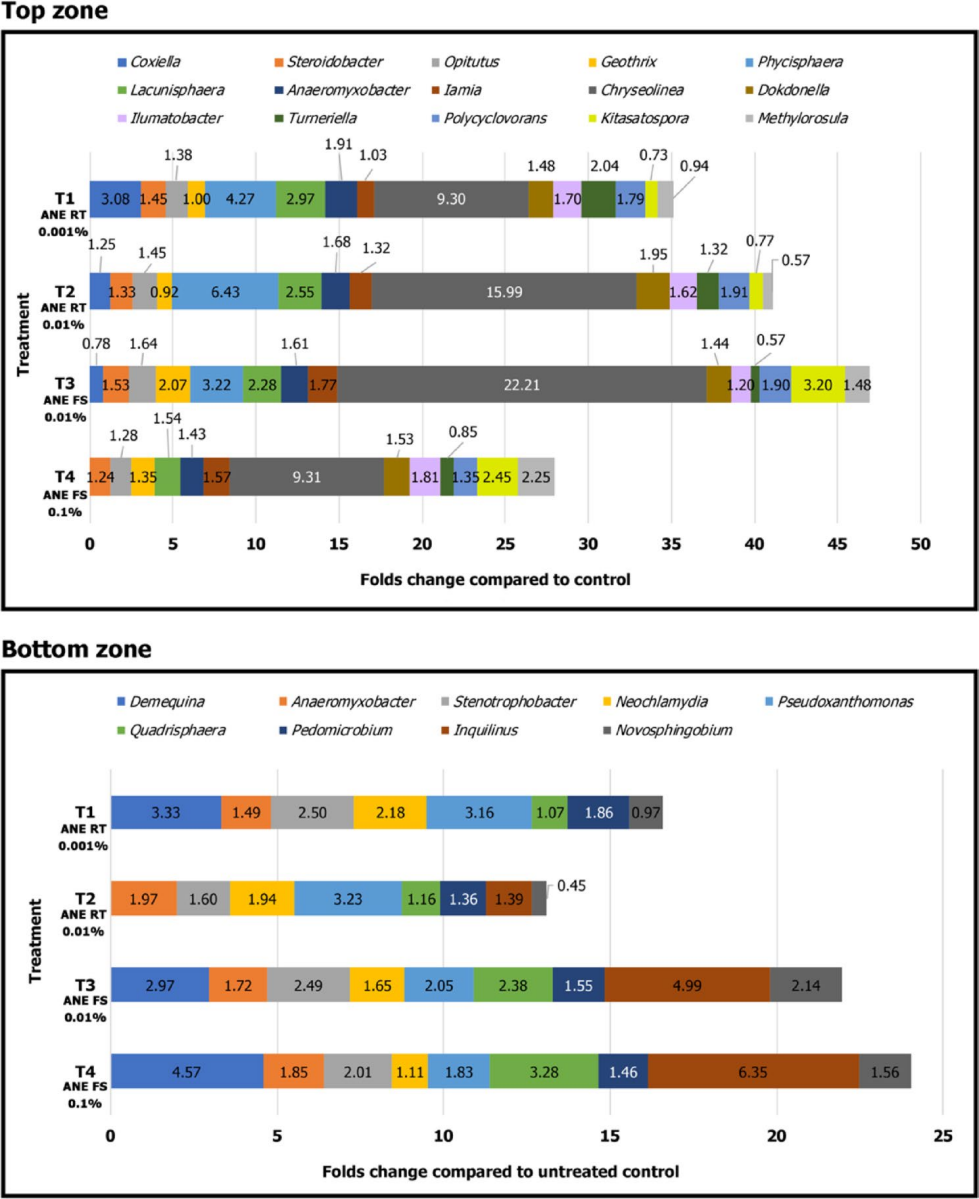
Folds change in the specific bacterial community in the maize, rhizosphere, soil treated with ANE. Data are represented as folds change compared to untreated control. T1 – Soil drench with 0.001% ANE; T2 - Soil drench with 0.01% ANE; T3 – Foliar spray with 0.01% ANE; T4 - Foliar spray with 0.1% ANE. Error bars indicate standard deviation obtained from four replicates per treatment. Analysis of variance was performed through DMRT with SPSS statistics (version 25, IBM). Means followed by common letters are not significantly different

The highest colony-forming unit (CFU) count was observed in the T2-ANE RT 0.01% (soil drench with 0.01% ANE), which was approximately 2-fold higher than that of the soil control (T5C-Soil) (Fig. 3). Next-generation sequencing (NGS) data analyses revealed only slight differences in the predominant bacterial community in the soil, but significant changes were noted in the secondary bacterial community, with a marked increase in a wide variety of bacterial genera in ANE-treated plants compared to the untreated control (Fig. 4). The top zone of the rhizosphere was particularly influenced by ANE treatment, exhibiting a multifold increase in bacterial reads compared to the untreated control.

The abundance of different bacterial genera varied across the treatments. Among these, the *Chryseolinea* genus showed the most pronounced response to ANE treatments, with its population in the rhizosphere soil increasing by approximately 22-fold under the T3-ANE FS 0.01% (foliar spray with 0.01% ANE) (Fig. 4, top panel). *Phycisphaera* sp. was the second most frequently observed bacterium in rhizosphere soil samples treated with ANE, with its highest population in the T2-ANE RT 0.01% (soil drench with 0.01% ANE), showing a 6.4-fold increase compared to the control (Fig. 4, top panel).

In addition, several other bacterial genera showed an increased relative abundance across all ANE treatments compared to the untreated control. These included: *Inquilinus* (~ 6.4-fold at T4-ANE FS 0.1%), *Novosphingobium* (~ 2.1-fold at T3-ANE FS 0.01%), *Turneriella* (~ 2-fold at T1-ANE RT 0.001%), *Kitasatospora* (~ 3.2-fold at T3-ANE FS 0.01%), *Lacunisphaera* (~ 3-fold at T1-ANE RT 0.001%), *Pseudoxanthomonas* (~ 3.2-fold at T2-ANE RT 0.01%), *Quadrisphaera* (~ 3.3-fold at T4-ANE FS 0.1%), *Stenotrophobacter* (~ 2.5-fold at T1-ANE RT 0.001%), *Demequina* (~ 4.6-fold at T4-ANE FS 0.1%) (Fig. 4; Table S2).

**Fig. 5:**
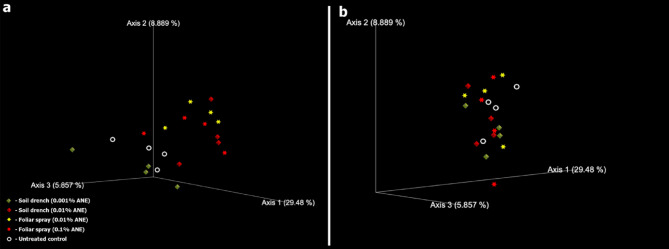
Weighted Unifrac of the bacterial community in the rhizosphere. (a) Top zone of the soil. (b) Bottom zone of the soil. The graph was produced by utilizing the Emperor ordination in Qiime2view

The Weighted UniFrac analysis revealed distinct clustering patterns for samples from the top zone. Rhizobacterial communities in the untreated samples were clearly separated from those in the soil-treated (0.01% ANE) and foliar-treated (0.1% ANE) samples (Fig. 5a). However, no distinct separation pattern was observed for the bottom zone samples (Fig. 5b). Overall, these results indicate that both foliar and soil drench ANE treatments significantly altered the structure of the rhizosphere bacterial community, particularly in the top zone.

### Analyses of root secreted compounds through LC-HRMS

**Fig. 6:**
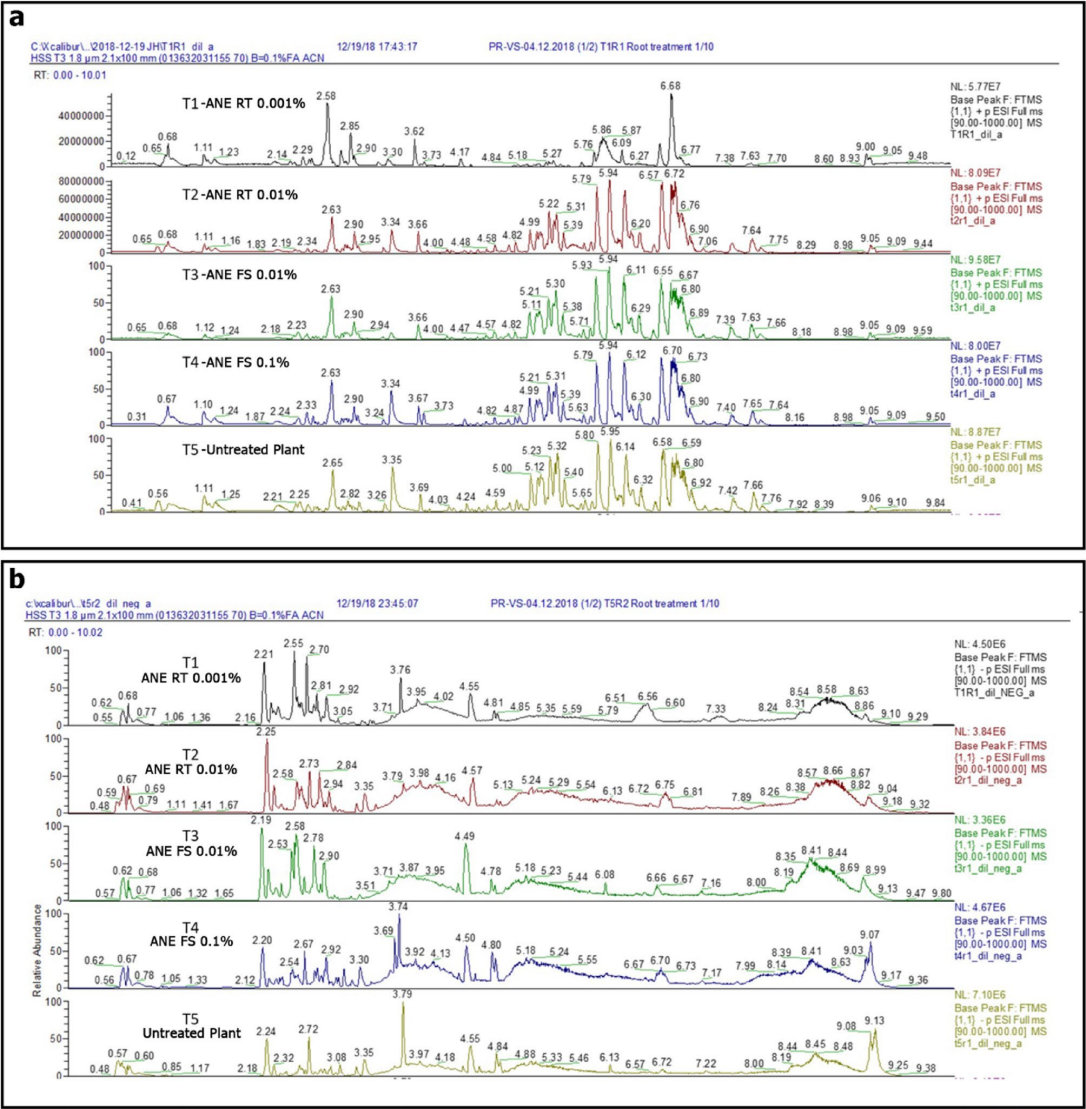
Representative LC-MS chromatograms of analysed root samples from maize plants treated with ANE. T1 – Root treatment with 0.001% ANE; T2 - Root treatment with 0.01% ANE; T3 – Foliar spray with 0.01% ANE; T4 - Foliar spray with 0.1% ANE; T5 – Untreated control. (a) Positive mode. (b) Negative mode

**Fig. 7:**
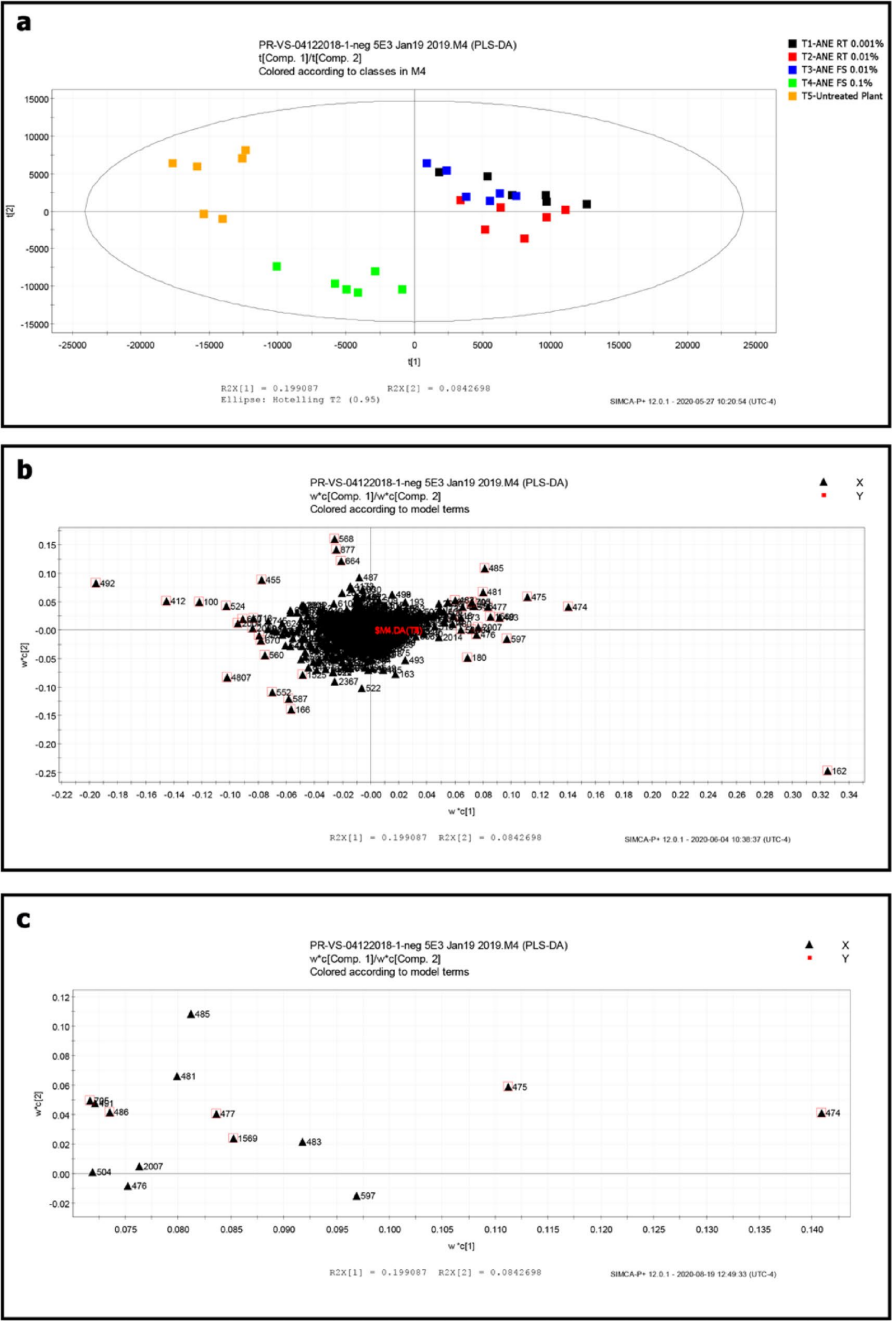
PLS-DA plots of the root extract from maize plants treated with ANE. (A) Score plot. (B) Loading plot. (C) Expanded loading plot showing tentative identification of benzoxazinoids (DIBOA, DIMBOA, MBOA, HDMBOA, and HMBOA). T1 – Root treatment with 0.001% ANE; T2 - Root treatment with 0.01% ANE; T3 – Foliar spray with 0.01% ANE; T4 - Foliar spray with 0.1% ANE; T5 – Untreated control. LC-MS data from negative ionization mode

Root extract, rhizosphere soil extract, and root exudate solution were analysed using LC-HRMS for untargeted metabolomics analysis. Among all the experiments, root extract samples showed higher degree of variation among the different ANE treatments (Fig. 6). LC-HRMS chromatograms were highly diverse between the treatments with differential peaks. Further multivariate data analysis and PLS-DA score plot revealed the root extract sample clustering into different groups that are diverse and were clearly separated from the control (Fig. 7A). No clear pattern of separation was observed in the PLS-DA score plots of root exudate and rhizosphere soil extracts (data not shown). In the loading plots (Fig. 7B and C) of root extract samples, the ions ID 474, 475, 1569, 477, 486, and 705 were tentatively identified as benzoxazinoids, based on accurate mass, fragmentation pattern, and literature reports [30, 31] (Table 1).

**Table 1:** Discriminative ions observed in root extracts

Ion ID	Retention time (min)	Accurate mass, m/z (difference from calculated mass)	Molecular formula	Fragment ion	Compound name (reference)
474	2.60	194.04543 ([M-H]^−^, −2.32 ppm) 196.06061 (MH^+^, 0.90 ppm)	C_9_H_9_NO_4_	138.05536 (C_7_H_8_NO_2_^−^)	2-hydroxy-7-methoxy-1,4-benzoxazin-3-one (HMBOA) (58)
475	2.21	180.02954 ([M-H]^−^, 3.84 ppm) 182.04486, (MH^+^, 0.42 ppm)	C_8_H_7_NO_4_	134.02414 (C_7_H_4_NO_2_^−^)	2,4-dihydroxy-1,4-benzoxazin-3-one (DIBOA) (58)
477	2.59	224.05592 ([M-H]^−^, −2.35 ppm) 226.07111 (MH^+^, 0.49 ppm).	C_10_H_11_NO_5_	194.04547 (C_9_H_8_NO_4_^−^)	2-hydroxy-4,7-dimethoxy-1,4-benzoxazin-3-one (HDMBOA) (59)
486	2.30	210.04045 ([M-H]^−^, −1.65 ppm) 212.05550 (MH^+^, 0.71 ppm)	C_9_H_9_NO_5_	195.01698 (C_8_H_5_NO_5_^−^)	2,4-dihydroxy-7-methoxy-1,4-benzoxazin-3-one (DIMBOA) (59)
705	2.60			179.02194 (C_8_H_5_NO_4_^−^)	HMBOA fragment
1569	2.55	164.03459 ([M-H]^−^, −4.43 ppm) 166.05000 (MH^+^, 0.79 ppm).	C_8_H_7_NO_3_	149.01093 (C_7_H_3_NO_3_^−^)	6-methoxy-benzoxazolin-2-one (MBOA) (58)

**Fig. 8:**
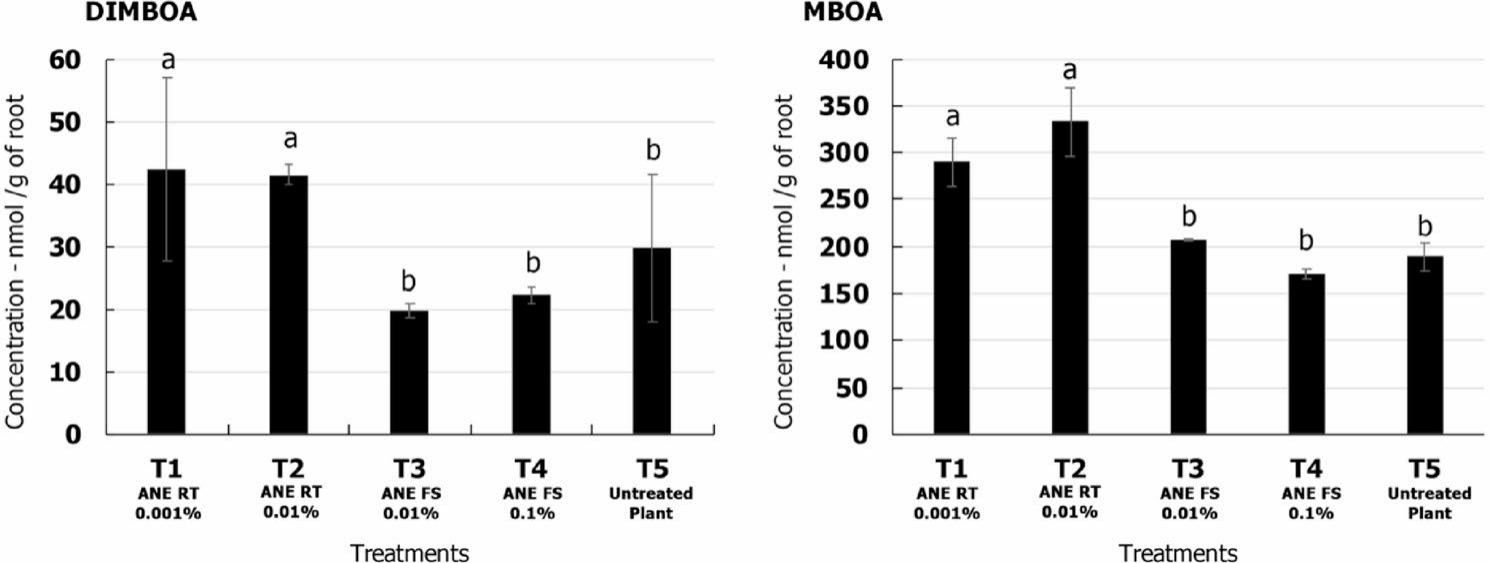
Quantitation of DIMBOA and MBOA in root samples of maize plants treated with ANE. Data are represented as µM/g of root sample. T1 – Root treatment with 0.001% ANE; T2 - Root treatment with 0.01% ANE; T3 – Foliar spray with 0.01% ANE; T4 - Foliar spray with 0.1% ANE. T5 – Untreated control. Error bars indicate standard deviation obtained from three replicates per treatment. Analysis of variance was performed through DMRT with SPSS statistics (version 25, IBM). Means followed by common letters are not significantly different

The presence of chemo-attractants including, DIMBOA and MBOA was further confirmed by comparing with reference standards. Even though these compounds were detected in all the experimental samples, only root extract samples showed consistent and reproducible results. The highest concentration of DIMBOA (42 nmol/g of root) and MBOA (333 nmol/g of root) was obtained at T2-ANE RT 0.01% (root treatment with 0.01% ANE) treatment which was ~ 1.4 folds and ~ 1.76 fold higher than that of untreated control (T5-Untreated plant), respectively (Fig. 8).

## Discussion

Results of this study add another facet to the mechanism of action of ANE, a seaweed-based plant biostimulant. Soil drenching or foliar sprays with ANE altered the rhizosphere microbiome and it appeared to be mediated by changes in the chemical composition of root exudates. The change in chemical composition of root exudate also affected the transcription of genes involved in chemotaxis and antimicrobial activity in *P. protegens* CHA0.

The chemotactic movement of bacteria towards root exudates has been well studied. In the present study, swarming motility results showed the movement of *P. protegens* (CHA0) towards root exudates of the ANE-treated maize plants (Fig. 1). The movement of the bacteria was much faster towards the root exudates obtained from root-treated plants with ANE (0.01%) compared to the untreated control. Moreover, the ANE treatment alone in the absence of root exudates did not have an effect. These results indicate that ANE treatment altered the composition of root exudate which in turn altered the motility of *P. protegens* CHA0 (Fig. 1). There are a number studies that have shown that components of root exudates affect chemotaxis of bacteria, malic acid and citric acid from watermelon root exudates induced the swarming motility of *Paenibacillus polymyxa* SQR-21. Furthermore, watermelon root treatment with organic acids enhanced the recruitment to rhizoplane [32]. Similarly, tomato root exudates (containing malic, citric, succinic acid, and fumaric acids) not only improved the growth of *Bacillus amyloliquefaciens* T-5 (up to two folds) but also increased the migration of the bacterial cells towards root exudates [46]. Moreover, *P. putida* and *P. fluorescens* exhibited chemotaxis towards soybean seed and root exudates [47, 48]. Other studies also demonstrated the induction of chemotaxis and biofilm formation in *Bacillus* species upon interaction with organic acids from the root exudates of cucumber, banana, and tomato [42, 49]. Components of A. nodosum like the fucose-rich polymers affected the swimming, swarming, and twitching motility of *P. aeruginosa* PA14 [50].

The chemotaxis pathway in *Pseudomonas* sp is complex and predominantly regulated by four gene clusters (I, II, IV, and V). Chemotaxis is influenced by three pathways including flagella-mediated pathways (*che* and *che2*) and a putative pili-mediated system [51]. The chemotaxis pathway *che* is essential for chemotaxis and the *che2* pathway is required for fine-tuning the chemotaxis. These pathways act through the clusters I, II, and V genes [52]. The pili-mediated pathway controls the twitching motility of the bacteria with cluster IV genes [53]. However, all the pathways share the homologous core genes namely, *cheA*,* cheB*,* cheR*,* cheW*,* cheY*,* cheV*, and *mcp*. The importance of these core genes has been reported in several studies. 3 A chemotactic mutant of *Ralstonia solancearum* lacking either *cheA* or *cheW* genes reduced the chemotaxis and virulence of the bacteria compared to wild type [54]. Chemotaxis gene *(cheA)* mutants of *Pseudomonas* species were defective in chemotaxis with reduced competitive colonization in response to tomato [55] and maize [56] root exudates. Plants communicate with rhizoplane microbes through a well-established chemical signaling pathway. Root exudate compounds from banana, induced the expression of antibiotic-producing genes (*ituA* and *bamD*) and biofilm formation genes (*epsD* and *yqxM*) in *B. amyloliquefaciens* NJN-6 [57]. In another study, bacteria exposed to artificial root exudates triggered the production of volatile metabolites which had a differential expression in the beneficial PGPR, *P. fluorescens* Pfo-1 [58]. Despite several studies on the effect of root exudates on chemotaxis and physiology of bacteria, the effect of treatment types (foliar/root drench) on root exudates alteration which in turn influenced the gene expression of the rhizosphere microbiome is still lacking. In the present study, root exudates from ANE-treated plants increased the transcript level of chemotaxis and antimicrobial genes in *P. protegens* CHA0.

In general, both soil drench and foliar spray with ANE induced transcript levels of several *P. protegens* CHA0 genes. The expression of *cheV*, *cheW*,* prnD*, *pvdS*, and *hcnA* was consistently up-regulated by the root exudates collected from T2-ANE RT 0.01% and T4-ANE FS 0.1% (Fig. 2). Contrary to this, the transcript level of *cheA* gene was only induced by T3-ANE FS 0.01%. Interestingly, ANE affected the transcript level of studied genes (without any root exudates), although to a lesser extent compared to root exudates treatments. Among the different ANE concentration, the higher concentration (T2C-ANE 0.01%) was found to be more effective as the expression of *cheV*, *cheW*,* prnD*, and *pvdS* were enhanced as compared to that of the control. These results suggest the direct and indirect potential of ANE in altering the expression level of the genes involved in chemotaxis and antimicrobial genes in *P. protegens* CHA0.

Earlier studies indicated that the CFU counts of bacteria and fungi in the ANE-treated soil increased in strawberry and carrot [59, 60]. A recent study demonstrated the efficiency of ANE in altering the rhizomicrobiome in pepper and tomato plants. Amplicon sequencing targeting fungal ITS and bacterial 16 S rRNA gene revealed significant differences in the diversity and relative abundance of rhizomicrobiome in response to ANE treatment [61]. Apart from ANE, other seaweed extracts have also been reported to influence microbial diversity either directly or indirectly. *Chondrus crispus* and *Sarcodiotheca gaudichaudi* directly declined the growth, biofilm formation capacity, and motility of *Salmonella enteritidis*. Furthermore, the expression of quorum sensing and pathogenesis associated genes of the bacterium were reduced by *C. crispus* and *S. gaudichaudi* [62]. In laying hens, feed supplementation with the red seaweeds including *Chondrus crispus* and *S. gaudichaudi* reduced *Salmonella enteritidis* population. Further, they also altered the relative abundance of the beneficial bacteria in the ceca [63]. Diet supplementation with *C. crispus* altered the colon microbiota in Sprague-Dawley rats; the population of beneficial *Bifidobacterium breve* was enhanced while that of pathogenic species including *Clostridium septicum* and *Streptococcus pneumonia* declined [64]. In another work, seed-applied biostimulants, comprising seaweed extract and phytohormones, increased the population of beneficial genera including *Chryseolinea*,* Quadrisphaera*,* and Turneriella* in the maize rhizosphere [65]. Findings of the present study were in line with the previous studies. In the present work, the bacterial community in the rhizosphere of maize plant was significantly increased by ANE application (Fig. 3). Similar to the gene expression results, the highest CFUs of culturable bacteria was observed in T2-ANE RT 0.01% and T4-ANE FS 0.1%. Interestingly, direct ANE treatments, T1C-ANE 0.001% and T2C-ANE 0.01% without any plants also increased the CFU of the culturable bacteria implying the direct and indirect effect of ANE in altering the CFU of culturable bacteria in the soil. Amplicon sequencing results further confirmed the shift in the population structure of rhizosphere bacteria (Fig. 4). The abundance of several notable genera with beneficial activity including, *Chryseolinea* (nitrogen cycling), *Pseudoxanthomonas* (nitrate and nitrite reduction), *Novosphingobium* (nitrogen-fixing), *Quadrisphaera* (antimicrobial), *Turneriella* (antimicrobial), *Kitasatospora* (synthesis of antifungal and nematicidal compounds) was enhanced by ANE treatment [65–68].

Chemical components of root exudates are known to play a significant role in plant-microbe interactions. Benzoxazinoids including, 2,4-dihydroxy-7-methoxy-2*H*−1,4-benzoxazin-3(4*H*)-one (DIMBOA) and 6-Methoxy-2-benzoxazolinone (MBOA) are major secondary defense metabolites in the root exudates of *Poaceae* [69]. DIMBOA from maize plants attracted *P. putida* to the rhizosphere which was attributed to the enhanced expression of bacterial mobility genes [13]. Using gene-specific mutants, benzoxazinoids were shown as a chemo-attractant of host-associated microbes in maize and pathogens inhibitor [70]. It is evident in the current study that ANE treatment altered the secretion of DIMBOA and MBOA in the maize roots (Figs. 6 and 7). In some studies, it was reported that MBOA concentration was higher in the root tissues rather than the root exudates itself due to absorption [71]. In maize, the distance between two plants affected the secretion of DIMBOA and MBOA. Closer planting improved the secretion of the compounds compared to the plants raised in distance. Suggesting that when the roots of different plants are in close contact the secretion of the compounds increased [72]. In the present study, the plants were raised single in containers that might have reduced the exudation quantity of the compounds into the root exudates. However, in the root tissues the concentration of the MBOA was significantly enhanced (≥ 2-fold) by in the treatment T2-ANE RT 0.01%, while that of DIMBOA did not change significantly (Fig. 8). DIMBOA undergo heterocyclic ring contraction and releases MBOA that are much more stable to be detected from the soil [73, 74]. In our experiment, DIMBOA and its breakdown product MBOA are detected in higher concentration in soil drenching with 0.01% ANE.

## Conclusion

Results of the present study concludes that both foliar and root treatment of maize plants with ANE resulted in direct and indirect effects. Root exudates from root-treated plants with 0.01% ANE (T2) enhanced the warming motility of *P. protegens* (CHA0). Root exudates from ANE treated plants resulted in the differential expression of genes related to chemotaxis (*cheA*,* cheW*,* cheV*), pyoverdine synthesis (*pvdS*), pyrrolnitrin production (*prnD*), and hydrogen cyanide synthesis (*hcnA*). Among all the treatments, root treatment with 0.01% ANE was the most effective in inducing the expression of genes related to chemotaxis and antimicrobial activity in *P. protegens* CHA0. ANE treatment significant effects and altered the structure of the rhizosphere bacterial community. The presence of chemo-attractants including, DIMBOA and MBOA were significantly higher in the root of plants root-treated with ANE.

## Supplementary Information

Below is the link to the electronic supplementary material.

Supplementary Material 1

## Data Availability

All data generated or analysed during this study are included in this manuscript and its supplementary information files.

## Abbreviations

ANE: Ascophyllum nodosum-based biostimulant
LC–HRMS: Liquid chromatography-high resolution mass spectrometry
PLS-DA: Partial least squares-discriminant analysis
DIMBOA: 2,4-dihydroxy-7-methoxy-1,4-benzoxazin-3-one
MBOA: 6-methoxybenzoxazolinone
EBIC: European Biostimulant Industry Council
FS: Foliar spray
RT: Root treatment
HS: Hoagland solution
CFU: Colony-forming unit
ANOVA: Analysis of Variance
