# Supplementary material for: Ascophyllum nodosum based plant biostimulant shapes the bacterial community in the rhizosphere of corn
Source: BMC Plant Biol. 2025 Oct 1;25:1263. doi: 10.1186/s12870-025-07270-7 (PMC12486656; doi:10.1186/s12870-025-07270-7)
Supplement: Supplementary file 1 — Supplementary Material 1 [file 12870_2025_7270_MOESM1_ESM.pdf]

Supporting information

Table S1. Primer pairs for qRT-PCR analyses of key genes in *P. protegens* (CHA0)

Function	Key gene	Primer sequences
Chemotaxis	*cheA1*	5′ GCCGGCGAGATTCTAGAGC 3′
		5′ AAGCACCACGTCCATCAGTTC 3′
	cheW	5′ AAGCAAGTGGTCGGGATCAT 3′
		5′ GTTCCGACCATTCCTCTTCG 3′
	cheV	5′ CTTTGTCTGTGGGGTGGTCA 3′
		5′ TGGCTTCCCAGTTCATGTTG 3′
Pyoverdine	*pvdS*	5′ GTCACGCCATCGACAAAGAA 3′
		5′ TCCATCCATTCGTTGAGCAG 3′
Pyrrolnitrin	*prnD*	5′ ACAAGCCGAAGGAGTTGACG 3′
		5′ CACTTGCCTTGCTCGTCGTA 3′
Internal control – RNA polymerase, beta prime subunit	*rpoC*	5′ ACCCAGGGCGAGAAGTACAA 3′
		5′ CCCGAGTCAGCCATCATGTA 3′

Table S2. Folds change in the bacterial community in the rhizosphere upon ANE treatment

S.No.	p-value	Genera	Folds change compared to control			
			T1	T2	T3	T4
1.	0.00	*Novosphingobium*	0.97	0.45	2.14	1.56
2.	0.01	*Inquilinus*	0.00	1.39	4.99	6.35
3.	0.02	*Kitasatospora*	0.73	0.77	3.20	2.45
4.	0.03	*Methylorosula*	0.94	0.57	1.48	2.25
5.	0.03	*Pedomicrobium*	1.86	1.36	1.55	1.46
6.	0.04	*Polycyclovorans*	1.79	1.91	1.90	1.35
7.	0.04	*Turneriella*	2.04	1.32	0.57	0.85
8.	0.07	*Quadrisphaera*	1.07	1.16	2.38	3.28
9.	0.07	*Ilumatobacter*	1.70	1.62	1.20	1.81
10.	0.07	*Pseudoxanthomonas*	3.16	3.23	2.05	1.83
11.	0.07	*Dokdonella*	1.48	1.95	1.44	1.53
12.	0.08	*Iamia*	1.03	1.32	1.77	1.57
13.	0.08	*Chryseolinea*	9.30	15.99	22.21	9.31
14.	0.08	*Neochlamydia*	2.18	1.94	1.65	1.11
15.	0.09	*Anaeromyxobacter*	1.91	1.68	1.61	1.43
16.	0.10	*Lacunisphaera*	2.97	2.55	2.28	1.54
17.	0.12	*Stenotrophobacter*	2.50	1.60	2.49	2.01
18.	0.13	*Phycisphaera*	4.27	6.43	3.22	0.00
19.	0.13	*Geothrix*	1.00	0.92	2.07	1.35
20.	0.14	*Opitutus*	1.38	1.45	1.64	1.28
21.	0.14	*Demequina*	3.33	0.00	2.97	4.57
22.	0.14	*Anaeromyxobacter*	1.49	1.97	1.72	1.85
23.	0.14	*Steroidobacter*	1.45	1.33	1.53	1.24
24.	0.15	*Coxiella*	3.08	1.25	0.78	0.00
